# A Recurrent Misdiagnosed and Maltreated Case of Keratosis Obturans

**DOI:** 10.1155/2019/9095747

**Published:** 2019-03-11

**Authors:** Hamoud Alarouj, Faisal AlObaid, Abdulmohsen K. AlBader, Mahmoud A. K. Ebrahim

**Affiliations:** ^1^Consultant Otolaryngology Head and Neck Surgery Farwaniya Hospital, Ministry of Health, Farwaniya, Kuwait; ^2^Consultant Otolaryngology Head and Neck Surgery and Head of ENT Department, Farwaniya Hospital, Ministry of Health, Farwaniya, Kuwait; ^3^Farwaniya Hospital, Ministry of Health, Farwaniya, Kuwait

## Abstract

Keratosis obturans is an obscure entity characterized by accumulation of desquamated keratinous material in the bony portion of the external auditory canal. Patients with this condition typically present with severe otalgia, conductive hearing loss, and global widening of the external auditory canal. We present a case of keratosis obturans that was misdiagnosed as impacted wax. The case was diagnosed and managed by microscope-guided examination with the patient under general anesthesia.

## 1. Introduction

Keratosis obturans (KO) is a rare condition of the external auditory meatus defined by accumulation of keratinaceous material in a lamellar arrangement that leads to dilation and blockage of the ear canal [1]. Although the condition was first described by Tonynbee in 1850, the term KO was first used by Wreden in 1874 who discriminated the condition from impacted ear wax [2, 3]. The characteristic clinical features include severe otalgia and hearing loss due to the collection of a desquamated epidermal plug in the ear canal [4]. The estimated incidence of KO is four to five patients per 1000 new otological cases [5]. Herein, we present a case of a patient initially diagnosed as having left impacted ear wax who underwent multiple failed trials of removal despite the use of alkaline ear drops. He was then referred to us and underwent microscope-guided examination under general anesthesia. He was diagnosed as having KO and managed accordingly.

## 2. Case Presentation

A 45-year-old male with a previous history of tympanoplasty and functional endoscopic sinus surgery with septoplasty 10 years earlier presented to the ear, nose, and throat (ENT) clinic with several months of left moderate-to-severe otalgia and a sensation of ear blockage in his left ear accompanied by ipsilateral hearing loss. He gave a history of multiple failed ear wax removal in his left ear that had been performed at several ENT clinics, despite the use of alkaline ear drops.

On examination, the patient was comfortable and afebrile, and his vital signs were stable. Otoscopic examination of the left ear revealed impacted left ear wax covering the tympanic membrane, which could not be assessed. Otoscopic examination of the right ear also demonstrated mild ear wax, and the tympanic membrane was unremarkable. Oropharynx examination was unremarkable, the lymph nodes of the neck were not palpable, and all cranial nerves were intact upon examination. Nasal endoscopy revealed no pathologies. Ear wax removal under suction was attempted and failed. Another trial of removal after using alkaline ear drops for several days was also attempted but was unsuccessful. However, the surgeon became suspicious that the patient had KO rather than impacted ear wax because the wax was thick, had the appearance of keratin plugs, and was hard to remove after several attempts, despite the use of ear alkaline drops.

**Figure 1:**
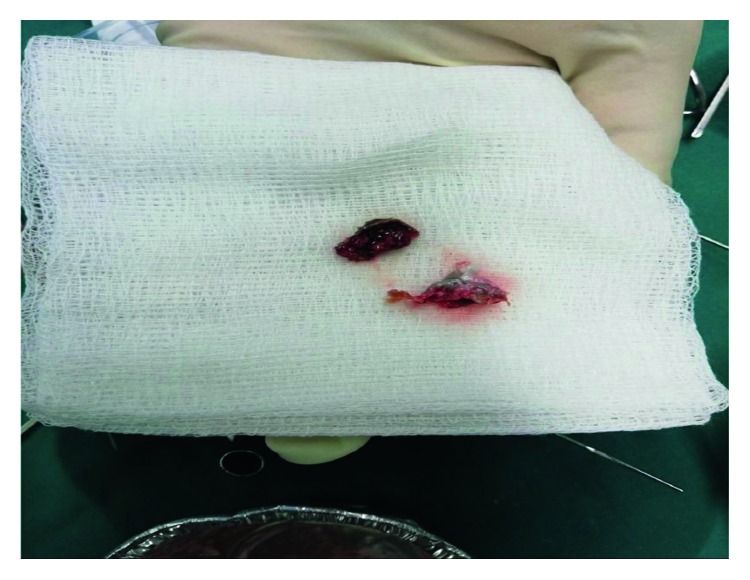
Postoperative procedure revealed thick keratinous plug mixed with wax.

Blood test results of the patient were within normal limits. The patient was planned for microscope-guided examination of the ears under general anesthesia. The examination revealed that the left ear was full of wax that was accumulating in the skin and contained a thick keratinous plug that had dilated the external auditory canal (EAC) with pockets and bone remodeling. Furthermore, the patient ear canal was circumferentially distended with a normal annulus. The tympanic membrane became visible and was intact. The keratinous plug was removed, and a diagnosis of KO was established (Figure 1). An ear pack was draped with antibiotics and placed in the left ear. The patient was extubated, shifted to the ward without any complications, and discharged the same evening with the ear pack, which was removed after 3 weeks in the outpatient clinic. The patient was started on ciprofloxacin ear drops and analgesia for 1 week.

**Figure 2:**
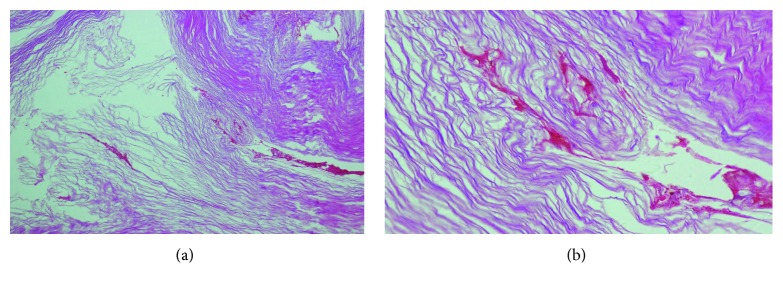
(a) Keratinous material (hematoxylin/eosin stain, ×100). (b) Acellular lamellated keratin flakes (hematoxylin/eosin stain, ×400).

In the follow-up, the ear pack was removed, his hearing returned to normal level, and the pain disappeared. Pathological analysis of the removed plug revealed acellular lamellated keratin flakes and keratinous material (Figures 2(a) and 2(b)), which confirmed our diagnosis.

## 3. Discussion

KO and external ear canal cholesteatoma were thought to represent the same disease, and the terms were used interchangeably to describe the accumulation of desquamated keratin in the bony part of the EAC until the late 20th century [6]. Piepergerdes et al. reviewed the literature in 1980 and was the first to state that KO and external ear canal cholesteatoma are two distinct diseases that are managed differently [7]. Although KO and external ear canal cholesteatoma are distinct diseases, they do have some overlapping characteristics that can make accurate diagnosis challenging [8]. KO typically manifests in a younger population and presents with acute conductive deafness, severe otalgia because of accumulation of keratin in the ear, widened ear canal, and a thickened tympanic membrane [9], whereas patients with external ear canal cholesteatoma present with otorrhea and chronic dull unilateral ache secondary to the invasion of squamous tissue into a localized area of periostitis in the canal [7]. Moreover, patients with KO can present with an extremely wide, circumferentially distended ear canal with an intact annulus “suspended in the air.” Our patient presented with a similar finding, but the annulus was not suspended in the air [10]. Otorrhea is a rare finding in KO, and bilateral occurrences are more common in children [11, 12]. Our patient had a previous history of sinusitis for which he was operated and treated.

The pathogenesis of KO remains obscure, although it has been associated with eczema, seborrhoeic dermatitis, or furunculosis. In addition, KO can occur with sinusitis or bronchiectasis (77% of juvenile and 20% of adult cases), which facilitates reflex sympathetic stimulation of the cerumen glands and consequent formation of an epidermal plug [9, 13]. Paparella and Shumrick proposed that excessive production of epithelial cells or faulty migration can result in an epidermal plug. In addition, circumferential widening of the body EAC may occur but with no evidence of osteonecrosis or bony sequestration, as found in EAC cholesteatoma [9]. These findings were consistent with those of our patient intraoperatively. A soft tissue plug in the bilateral external ear canal with evidence of ballooning of the osseous part are the common findings in computed tomographic scans of patients with KO [4].

There are two types of KO: inflammatory or silent type [5]. It has been proposed that the mechanism underlying the inflammatory type is an acute infection, such as viral infection, through which the epithelial migration can be transiently changed by the inflammatory process. Removal of the keratin can cure the inflammatory type. The silent type is caused by abnormal separation of the keratin leading to the continuous progression of the disease, even after the first removal; thus, continuous regular aural toileting is required to treat the disease [4]. In addition, patients with KO not associated with inflammation of the canal skin will need lifelong periodic aural toileting because of the local metabolic deficiency that affects the normal migratory mechanism [14]. Topical medication and frequent aural toileting are usually needed to remove the keratin plug, which for noncooperative patients can be performed by administering general anesthesia. For refractory cases of the disease, split skin graft and canaloplasty method have been used to treat these cases [15]. Our patient presented with the inflammatory type for which he was managed with a single keratin plug removal. A previous study demonstrated that complications of untreated KO include sensorineural hearing loss, dehiscence of the tegmen tympani, and facial palsy [16].

Several different types of ear drops are available to apply for wax removal and softening, which include oil-based compounds (e.g., olive oil), water-based compounds (e.g., sodium bicarbonate or water itself), and nonwater, non-oil-based solutions (e.g., hydrogen peroxide-urea compound) [17]. A recent Cochrane review found no significant difference between the different types of ear drops, as they were all equally effective in treatment [17]. Side effects of sodium bicarbonate ear drops include ear dryness and a mild stinging sensation, whereas an unpleasant taste, temporary bubbling sensation, increased pain in the ear, temporary loss of hearing, dizziness, and tinnitus were side effects of nonwater-non-oil ear drops (e.g., urea-hydrogen-peroxide drops). Multiple types of olive oil ear drops exist; some patients may experience a tingling or stinging sensation or mild temporary deafness. A damaged tympanic membrane, pain, discharge, inflammation, infection, and tinnitus within 2-3 days of irrigation are contraindications for hydrogen peroxide ear drops. On the contrary, the contraindications for oil ear drops are allergy, perforated tympanic membrane, ear infection, outer ear eczema, or seborrhoeic dermatitis. No contraindications are known for sodium bicarbonate ear drops [18]. However, the tolerance of patients to the side effects of different ear drops was assessed in a recent study showing no significant difference between them [17]. Needless to say, the ear drops used by our patient were an alkaline-based ear drop solution.

It is challenging to discriminate between KO and impacted wax at the initial presentation. The diagnosis of KO, as a clinical entity, is often reached only after attempting to remove the accumulated desquamated keratin from deep in the ear canal, which causes intense pain and the appearance of a silvery white peripheral matrix. Bleeding may occur as a result of the stripping or peeling off of the matrix, which may be the result of neovascularization; the formation of new capillaries around the matrix is most likely the outcome of inflammation or irritation of the neighboring skin of the bony canal [19]. We believe that if a patient is encountered with thick impacted wax showing unusual characteristics that cannot be removed after several attempts on different occasions, other pathologies should be considered. Careful otoscopic examination should be performed for all patients to assess the ear canal and features of the wax. In our case, the surgeon performed a careful otoscopic and visual examination and determined that the features suggested KO. The diagnosis and management of KO are different from other possible conditions, such as external ear canal cholesteatoma.

## 4. Conclusion

KO is regarded as a benign condition, but it can result in serious complications. As seen in our case, the condition can be misdiagnosed and maltreated; thus, it is essential for early detection and correct management to prevent complications and relieve symptoms.
